# Physical Health Simulation Based Education (SBE) for psychiatrists in the first COVID-19 wave: improving the competence and confidence of the medical workforce

**DOI:** 10.1192/bjo.2021.414

**Published:** 2021-06-18

**Authors:** Craig McEwan, Richard Kerslake, Michael Hobkirk

**Affiliations:** Sussex Partnership NHS Foundation Trust (SPFT)

## Abstract

**Aims:**

At the start of the COVID-19 pandemic there was significant uncertainty for the NHS and it's workforce. Within psychiatry, there was an expectation that junior doctors would be redeployed, with senior psychiatrists stepping down to cover physical health and on-call duties.

Senior leadership in mental health trusts were also preparing for COVID-19 outbreaks on psychiatric wards and were developing strategies for managing a novel illness with a poorly understood clinical course. Many psychiatrist expressed anxieties around their competency in assessing and managing acutely physically unwell patients in a mental health setting.

This project aimed to improve confidence of psychiatrists in core physical health competencies through devising and delivering an evolving SBE package.

**Method:**

Sussex Partnership Foundation Trust redeployed two higher trainees from their simulation faculty to work full time on developing a SBE package. This was requested by senior leadership to deliver training about assessing and managing physically unwell patients in the context of COVID-19. This training was devised as a 90 minute didactic lecture following by 90 minutes of SBE.

This was delivered at 6 sites through 10 opt-in sessions available to all doctors in the trust over 4 weeks. Pre and post-course questionnaires were given to all participants to measure the effect.

**Result:**

102 medical staff attended the SBE workshops. Feedback was completed by 93 (91%) doctors prior to the course and 97 (95%) post. Before the workshop, 33% did not feel they had a structured approach for assessing an acutely unwell patient, which reduced to 0% after completing the course.

On a 5-point Likert scale, confidence in managing COVID-19 symptoms increased from 2.54/5 to 4.07/5 overall with 89% of doctors feeling “confident” or “very confident”. There were similar increases in confidence in managing critically unwell patients (2.7/5 pre; 3.95/5 post) and in identifying alternative causes for acutely unwell patients (2.63/5 pre; 4.02/5 post).

**Conclusion:**

This project demonstrates that SBE is an effective way to rapidly develop effective interventions for the medical workforce, increasing confidence in the face of significant uncertainty and reducing anxiety within the system to meet the learning needs identified by medical leadership.

As part of this project Sussex Partnership Medical Education freely shared the workshop materials, which were later adopted and used by psychiatry departments internationally.

